# Bri2 BRICHOS domain inhibits IAPP amyloid formation and improves beta cell function in stem cell-derived islets under metabolic stress

**DOI:** 10.1007/s00125-025-06582-5

**Published:** 2025-11-10

**Authors:** Jing Cen, Anja Ivis, Svitlana Vasylovska, Kina Adjieva, Robin S. Lindsay, Gunilla T. Westermark, Joey Lau

**Affiliations:** Department of Medical Cell Biology, Uppsala University, Uppsala, Sweden

**Keywords:** BRICHOS, Diabetes, IAPP, Islet amyloid, SC-islets, Stem cell-derived islets

## Abstract

**Aims/hypothesis:**

Accumulation of islet amyloid polypeptide (IAPP) and amyloid formation is associated with beta cell dysfunction and cell death in human islets and may also contribute to graft failure post stem cell-derived islet (SC-islet) transplantation. The BRICHOS domain, a secretory peptide proteolysed from the Bri2 protein, possesses chaperone activity and has been shown to inhibit fibril formation of amyloid β-peptide in the brain and IAPP in human islets. In this study, we aimed to evaluate amyloid formation in SC-islets in vitro, as well as assess the role of Bri2 BRICHOS on amyloid formation and beta cell function.

**Methods:**

Human SC-islets were used as an in vitro model to explore the accelerated amyloid formation and to investigate the role of Bri2 BRICHOS via adenovirus-transduced overexpression. SC-islets were cultured under normal glucose conditions or metabolic stress-like conditions. Subsequently, amyloid formation was determined by staining with the amyloid-specific ligand pentameric formyl thiophene acetic acid and transmission electron microscopy. Beta cell function was assessed by static glucose-stimulated insulin secretion and insulin content. The presence of relevant proteins was evaluated by immunostaining and confocal microscopy. The mRNA expression profile of genes of interest was evaluated by qRT-PCR.

**Results:**

We showed that IAPP is colocalised with insulin in SC-islet beta cells and, like human islets, SC-islets can develop amyloid under metabolic stress in vitro. Amyloid formation was increased and beta cell function was impaired in SC-islets under metabolic stress and overexpression of the Bri2 BRICHOS domain in SC-islets effectively prevented amyloid formation and partially protected beta cell function. The accentuated endogenous gene expression of *ITM2B*, *ADAM10* and *IAPP* in SC-islets under the same glucose-induced metabolic stress condition was not affected by the overexpression of the Bri2 BRICHOS domain.

**Conclusions/interpretation:**

Our findings suggest that the molecular chaperone Bri2 colocalises with IAPP and insulin in SC-islet beta cells. The folding assistance has been ascribed to the BRICHOS domain in Bri2 and viral overexpression of the BRICHOS domain can prevent the formation of cytotoxic IAPP amyloid and improve beta cell function in SC-islets exposed to metabolic stress. A comprehensive analysis of SC-islet functionality excludes beta cell impairment as a cause for amyloid reduction but supports the protection against IAPP amyloid.

**Graphical Abstract:**

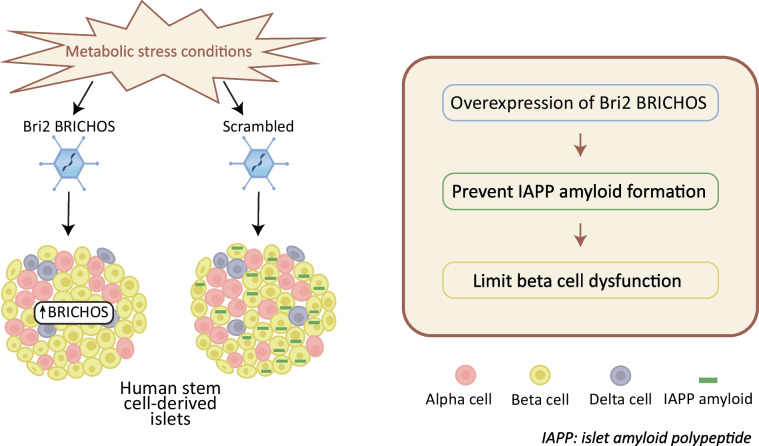

**Supplementary Information:**

The online version of this article (10.1007/s00125-025-06582-5) contains peer-reviewed but unedited supplementary material.


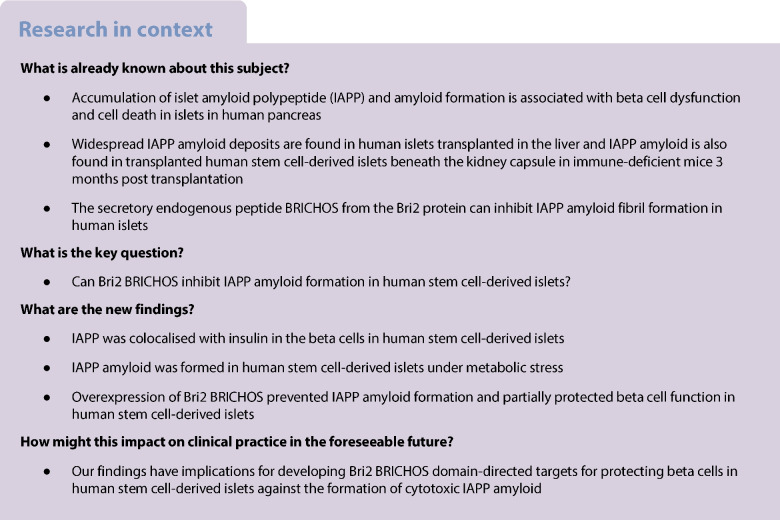


## Introduction

Islet amyloid formation is considered an important pathological characteristic of pancreatic islets in individuals with type 2 diabetes [1–3], while it can also be identified in some individuals with type 1 diabetes [4, 5] or less commonly in non-diabetic individuals [6, 7]. The main constituent of the deposited amyloid in human islets is the polypeptide hormone, islet amyloid polypeptide (IAPP), which is secreted together with insulin from beta cells [8, 9]. Human IAPP has amyloidogenic properties and the accumulation of IAPP amyloid in human islets can lead to beta cell dysfunction and cell death [3, 10, 11], which is one of the factors that contribute to graft failure post islet transplantation [10, 11].

The generation of stem cell-derived islets (SC-islets) from human pluripotent stem cells (hPSCs) holds great potential to provide an unlimited source of insulin-producing beta cells for the treatment of diabetes [12–14]. However, like in human islets, beta cells in SC-islets express IAPP [14, 15]. In an in vivo study, amyloid developed in implants from 20 weeks post transplantation of human stem cell-derived pancreatic endoderm placed in the subcutis of immune-compromised mice [16]. Recently, we confirmed the development of amyloid at 3 months following transplant of human SC-islet grafts beneath the renal capsule in immunodeficient mice [15]. These results are consistent with previous findings where amyloid develops in human islets transplanted into mice at 2 weeks post transplantation [17] and in intraportal-vein-transplanted human implants recovered after 2 years [10]. The formation of amyloid can be extensive and deposits in the transplanted SC-islets will impair the beta cell function. Thus, it is important to explore the factors that can inhibit IAPP amyloid formation in SC-islets to prevent beta cell dysfunction in the long term.

Molecular chaperones can assist protein folding and counteract misfolding during protein synthesis [18]. Bri2, also known as integral membrane protein 2B (ITM2B), is a multidomain transmembrane protein expressed in a variety of tissues [19, 20]. In human islets, Bri2 colocalises with IAPP intracellularly in beta cells [21]. The Bri2 protein contains a 100 residue-long BRICHOS domain attributed to a chaperone-like function and has been shown to assist folding and inhibit fibril formation of amyloid β-peptide and IAPP, associated with Alzheimer’s disease and type 2 diabetes, respectively [21, 22]. In in vitro studies, the BRICHOS domain interacts with IAPP and drives aggregation to the formation of non-toxic amorphous aggregates. Furthermore, the downregulation of Bri2 expression with siRNA in EndoC-βH1 cells made cells more vulnerable to metabolic stress, a disadvantage ameliorated by simultaneous overexpression of the BRICHOS domain [21]. However, the effect of the Bri2 BRICHOS domain on beta cell function has not yet been studied. Therefore, in this in vitro study, we aim to investigate the circumstances under which IAPP amyloid forms in SC-islets and whether overexpression of the Bri2 BRICHOS domain affects IAPP amyloid formation and beta cell function in SC-islets.

## Methods

### Human embryonic stem cell differentiation and human islet culture

Human embryonic stem cells (hESCs) (H1 cell line, no mycoplasma contamination) (WiCell Research Institute, WI, USA) were differentiated into SC-islets using a seven-stage differentiation protocol as previously described [14]. Isolated human islets from brain-dead, non-diabetic donors were used for comparison studies (see the human islet checklist in the electronic supplementary material [ESM]) and were generously provided by the Nordic Network for Clinical Islet Transplantation (Uppsala University Hospital, Uppsala, Sweden). Ethical permission to use human islets has been obtained from the Regional Ethical Review Board in Uppsala, Sweden (Regionala etikprövningsnämnden, Uppsala, Sweden). Human islets were cultured in CMRL1066 medium (Gibco, MA, USA) supplemented with 5.5 mmol/l glucose, 10% vol./vol. FBS (Gibco), 100 units/ml penicillin–streptomycin (Sigma-Aldrich, MO, USA) and 1% vol./vol. glutamine (Sigma-Aldrich) at 37°C in humidified air containing 5% CO_2_. Human islets from ten donors (ESM Table 1) were used in this study (mean ± SD age 62.7 ± 2.7 years; *n* male/*n* female 5/5; mean ± SD BMI 26.9 ± 1.3 kg/m^2^; mean ± SD HbA_1c_ 39.9 ± 1.4 mmol/mol [5.8 ± 2.3%]).

During the experiment, SC-islets or human islets were cultured in the respective culture medium, without or supplemented with 20 mmol/l glucose, 1.5 mmol/l sodium palmitate (Sigma-Aldrich) or a combination of 20 mmol/l glucose (Sigma-Aldrich) and 1.5 mmol/l sodium palmitate (Sigma-Aldrich) for the duration specified. Sodium palmitate was prepared as described previously [23]. The culture medium was changed every 2–3 days. The SC-islets and the human islets were distributed equally (regarding size) into each group (e.g. control vs experimental group). SC-islets, or human islets that have fused during culture and become very large, were not included in experiments due to the presence of necrotic cores.

### Overexpression of Bri2 BRICHOS domain in SC-islets

The adenovirus Ad-BRICHOS was used for SC-islet transduction, resulting in the expression of a signal peptide with 23 residues linked to residues 90–263 of Bri2 [21]. A non-protein-expressing adenovirus was used as control (Ad-control) [24]. SC-islets were transduced with Ad-BRICHOS or Ad-control for 30 h, followed by culturing in 5.5 mmol/l glucose (G5.5 mmol/l) or 20 mmol/l glucose (G20 mmol/l) for 7 or 10 days.

### Pentameric formyl thiophene acetic acid staining of amyloid in human islets and SC-islets

The presence of amyloid in human islets and SC-islets was visualised with the luminescent conjugated oligothiophene pentameric formyl thiophene acetic acid (pFTAA) [25, 26]. After culture, human islets or SC-islets were incubated with culture medium supplemented with 7.5 μmol/l of pFTAA (kindly provided by Peter Nilsson, Linköping, Sweden) for 2 h, rinsed with PBS and placed in a glass-bottomed optical dish (IBIDI, WI, USA) with PBS, and then imaged with confocal microscopy using excitation λ 488 nm and z-stack protocol (Zeiss LSM780, Oberkochen, Germany). Images were analysed using ImageJ 1.53q [27], and the amount of amyloid was calculated and presented as the ratio of pFTAA-positive area to the total area of human islets or SC-islets.

### Glucose-stimulated insulin secretion from SC-islets

KRBH buffer supplemented with 0.1% BSA and adjusted to pH 7.4 was used to perform the static glucose-stimulated insulin secretion (GSIS) studies in SC-islets at 37°C. SC-islets in duplicates (around ten SC-islets each) from different culture conditions were pre-incubated in KRBH buffer with 2.8 mmol/l glucose for 90 min, followed by incubation at low glucose concentration (2.8 mmol/l) for 60 min and then with high glucose concentration (16.7 mmol/l) for 60 min. Supernatant fractions from low and high glucose concentrations were collected for insulin secretion measurement. SC-islets after GSIS were homogenised by sonicating in 100 µl of redistilled H_2_O. An aliquot was used for DNA content measurement for sample comparisons. From the sonicated sample, 25 µl solution was mixed with 62.5 µl of 95% acid ethanol and then stored at −20°C until measurement of insulin content. Insulin concentration was measured by Insulin ELISA Assay Kit (Mercodia, Uppsala, Sweden). DNA content was measured by the PicoGreen dsDNA quantification assay (Invitrogen, CA, USA).

### Oxygen consumption rate in SC-islets

Oxygen consumption rate (OCR) and extracellular acidification rate (ECAR) of SC-islets were determined by Extracellular Flux Analyzer XF^e^96 (Agilent technologies, CA, USA) as previously described [23]. SC-islets (eight or nine SC-islets/well, four or five replicates for each culture condition) were placed into the XF^e^96 cell culture microplate pre-coated with Laminin 521 (10 μg/ml; BioLamina, Stockholm, Sweden) and incubated in assay medium (Agilent technologies) with 2.8 mmol/l glucose for 2.5 h at 37°C in air. The basal OCR at 2.8 mmol/l glucose was then measured, followed by sequential injection of 20 mmol/l glucose and different mitochondrial respiration chain inhibition compounds. ECAR was analysed in parallel.

### Beta cell apoptosis in SC-islets

SC-islets from different culture conditions were made into single-cell suspensions using non-enzymatic cell dissociation buffer (Thermo Fisher Scientific, Eugene, USA) and stained for live/dead discrimination using LIVE/Dead Fixable Aqua (Thermo Fisher Scientific), followed by staining with the early apoptotic marker Annexin V (Thermo Fisher Scientific) [28–30]. Following fixation and permeabilisation using the Foxp3 transcription factor buffer (Thermo Fisher Scientific), cells were stained intracellularly with an anti-insulin antibody (Cell Signaling Technology, Danvers, USA), followed by flow cytometry using a Northern Lights cytometer equipped with three lasers (Cytek, Fremont, USA). Analysis was performed using FlowJo software v10.10 (BD Bioscience, Franklin Lakes, USA).

### Immunofluorescence

SC-islets fixed with 4% paraformaldehyde for 20 min were washed with 0.1 mol/l phosphate buffer with 0.15 mol/l NaCl pH 7.4 (PBS), permeabilised with 1% Triton X-100 in PBS (Sigma, MO, USA) for 60 min at room temperature and blocked overnight at 4°C in PBS with 0.3% Triton X-100 and 5% donkey serum (Jackson ImmunoResearch, PA, USA). Afterwards, SC-islets were incubated with primary antibodies overnight at 4°C, rinsed and incubated with secondary antibodies at room temperature for 1 h. Primary and secondary antibodies were diluted in PBS with 0.3% Triton X-100 and 5% donkey serum. After rinsing, SC-islets were mounted with a fluorescence mounting medium (Dako, CA, USA). Images were acquired using the confocal microscopy (Zeiss LSM780).

Primary antibodies were rabbit anti-ITM2B/Bri2 (1:1000) (HPA029292, Sigma-Aldrich), rabbit anti-IAPP (1:250) [31], guinea pig anti-human insulin (1:300) (Fitzgerald, Acton, MA, USA) and mouse anti-glucagon with Alexa Fluor 488-conjugated (1:150) (Invitrogen). Secondary antibodies were Alexa Fluor 594 donkey anti-guinea pig (1:300) (Jackson ImmunoResearch) and Alexa Fluor 647 donkey anti-rabbit (1:300) (Jackson ImmunoResearch). DAPI was used to stain the nuclei.

### Transmission electron microscopy

Five SC-islets from each culture condition were chemically fixed with 2.5% glutaraldehyde (EM-grade, Sigma-Aldrich) in PBS (pH 7.4) at room temperature for 2 h. After rinsing in PBS, the SC-islets were dehydrated with increasing concentrations of ethanol and embedded in EPON (Polyscience, Germany). Ultrathin sections placed on 200 mesh Cu-grids were contrasted with 2% uranyl acetate and Reynolds lead citrate. The material was analysed in a Tecnai G2 transmission electron microscope (FEI, Thermo Fisher).

### Quantitative reverse transcription PCR (qRT-PCR)

Total RNA of human islets and SC-islets was extracted using RNeasy Plus Micro Kit (Qiagen, Hilden, Germany), and cDNA was synthesised using the same amount of RNA of each sample and reverse transcribed using SuperScript First-Strand Synthesis SuperMix (Invitrogen) according to the manufacturer’s protocol. Quantitative real-time PCR (qPCR) was performed using PowerUp SYBR Green Master Mix (Applied Biosystems) on QuantStudio 5 Real-Time PCR Systems (Applied Biosystems). The relative gene expression levels between human islet and SC-islet cells were calculated by subtracting the geometric average C_t_ value of endogenous control *ACTB* and *RPS7*, followed by calculating with the ${2}^{{-\Delta \text{C}}_{\text{t}}}$ method. Relative gene expression in SC-islet experiments with Bri2 BRICHOS domain overexpression was calculated by subtracting the geometric average C_t_ value of endogenous control *GUSB* and *RPS7* and further expressed with the ${2}^{{-\Delta \Delta \text{C}}_{\text{t}}}$ method. Primers were synthesised by Eurofins Genomics (Ebersberg, Germany) and the sequences are listed in ESM Table 2.

### Exploring online RNA-seq database of human islets

Islet Gene View (IGW) platform (https://mae.crc.med.lu.se/IsletGeneView/) is a web tool based on the RNA-seq and genome-wide genotyping in human islets from 188 donors (155 without and 33 with type 2 diabetes) [32]. IGW was scrutinised for data on the expression of *ITM2B* in human islets.

### Data analysis

Data were analysed using GraphPad Prism10 (GraphPad software). The results generated from biological replicates were expressed as a mean of independently repeated experiments ± SEM. For the comparison of two groups, the two-tailed Student’s *t* test was used, and for the comparison of several groups, one-way ANOVA followed by the Holm–Sidak or Tukey multiple comparison tests was used for normally distributed data and Dunn multiple comparison tests for non-normally distributed data. A *p* value <0.05 was considered statistically significant. Data are available on request from the authors.

## Results

### IAPP expression in human islets and SC-islets

**Fig. 1:**
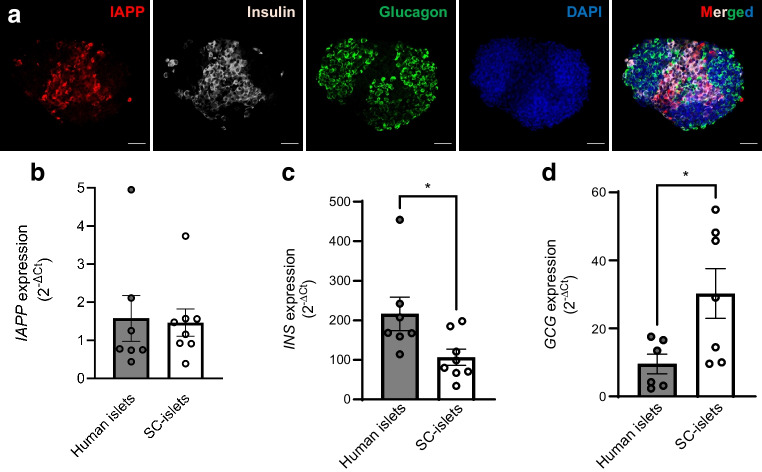
IAPP expression in human islets and SC-islets at stage 7, week 6. (**a**) SC-islets were differentiated from H1 cells using a seven-stage differentiation protocol. The expression and location of IAPP in SC-islets were confirmed by immunostaining and representative images are presented (IAPP, red; insulin, grey; glucagon, green; and DAPI, blue). Scale bar, 50 µm, *n*=12 SC-islets from three different batches of differentiation. (**b**–**d**) mRNA expression of genes of interest, including *IAPP* (**b**), *INS* (**c**) and *GCG* (**d**), was determined in human islets and SC-islets by qRT-PCR and the results were first normalised by using the geometric average C_t_ value of two endogenous controls (*RPS7* and *ACTB*) and further calculated with the ${2}^{{-\Delta \text{C}}_{\text{t}}}$ method. Results are expressed as mean ± SEM of *n*=6–7 human islet donors or 7–8 different batches of SC-islet differentiations. *p < 0.05 as indicated

To ensure the expression and distribution of IAPP at the later stage of differentiation, SC-islets at stage 7, week 6, were picked and examined. Immunostaining confirmed IAPP expression in SC-islets and that the peptide colocalised with insulin-stained beta cells (Fig. 1a). IAPP reactivity was absent in glucagon-positive alpha cells. There was no difference in the gene expression of *IAPP* between SC-islets and human islets (Fig. 1b). However, the expression of the *INS* gene in SC-islets reached only 50% of the level detected in human islets (Fig. 1c). In contrast, the expression of *GCG* in SC-islets was twice that of the level detected in human islets (Fig. 1d). The mRNA expression of these genes is in line with the RNA-seq results obtained from the comparison of SC-islets with human islets [15]. The differences in insulin and glucagon gene expression between SC-islets and human islets may depend on expression levels or indicate that the proportion of insulin-secreting beta cells and glucagon-secreting alpha cells differ between the differentiated SC-islets and isolated human islets.

### Amyloid formation was triggered in SC-islets under metabolic stress

**Fig. 2:**
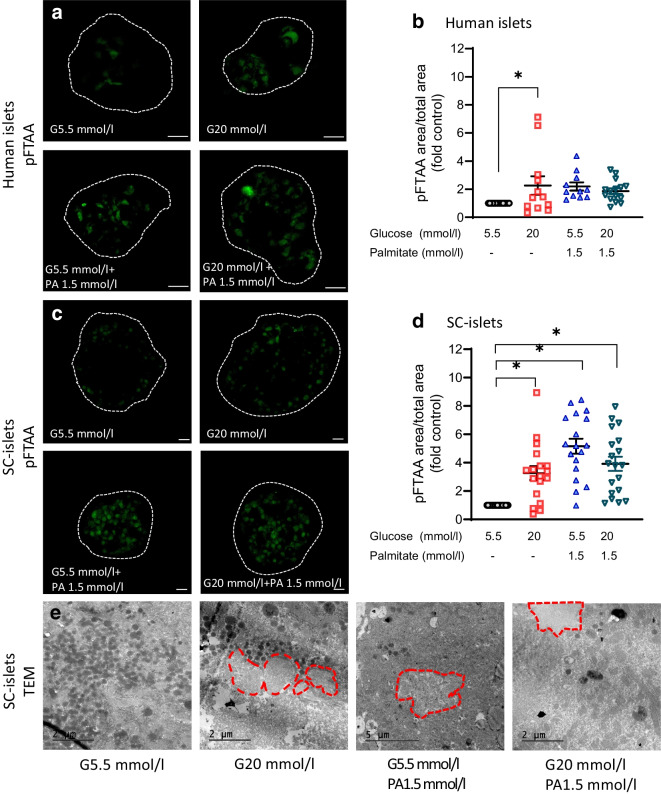
Amyloid formation in human islets and SC-islets in vitro under metabolic stress. Human islets or SC-islets were exposed to 5.5 mmol/l glucose (G5.5 mmol/l) or metabolic stress conditions, including 20 mmol/l glucose (G20 mmol/l), 1.5 mmol/l sodium palmitate with 5.5 mmol/l glucose (G5.5 mmol/l, PA 1.5 mmol/l), or a combination of 20 mmol/l glucose and 1.5 mmol/l sodium palmitate (G20 mmol/l, PA 1.5 mmol/l) for 14 days. After culture, human islets or SC-islets were stained with pFTAA and imaged by confocal microscopy. (**a**, **b**) Representative pFTAA staining images from human islets are presented (**a**) and the ratio of pFTAA-positive area to the total human islet area is shown (**b**). (**c**, **d**) Representative pFTAA staining images from SC-islets are presented (**c**) and the ratio of pFTAA-positive area to the total SC-islet area is shown (**d**). The white dashed lines denote the boundaries of human islets or SC-islets. Scale bar, 20 µm. (**e**) SC-islets were evaluated by transmission electron microscopy and representative images are presented. The red dashed lines denote the formation of amyloid in SC-islets. The results are expressed as the fold of the respective G5.5 mmol/l group and shown as mean ± SEM; *n*=total of 11–15 human islets from three donors (3–5 human islets per donor) and *n*=total of 18–22 SC-islets from four different batches of differentiations (4–5 SC-islets per differentiation). *p < 0.05 as indicated

Expression of IAPP and insulin are regulated in parallel [33], and prolonged beta cell stress (e.g. high concentrations of glucose) results in a simultaneous increase in insulin and IAPP synthesis, which can lead to the formation of amyloid in isolated islets from human IAPP transgenic mice [34]. In this study, the amyloid-specific ligand pFTAA was used for amyloid detection [25, 26]. Culture of human islets in 20 mmol/l glucose for 14 days resulted in the development of almost double the amount of amyloid compared with the amyloid load detected in the human islets exposed to 5.5 mmol/l glucose (Fig. 2a, b). When human islets were cultured in 1.5 mmol/l sodium palmitate or a combination of 20 mmol/l glucose and 1.5 mmol/l sodium palmitate, only a minor increase in amyloid formation was observed compared with islets cultured in 5.5 mmol/l glucose (Fig. 2a, b).

Interestingly, we found that amyloid formation in SC-islets was significantly triggered by all three metabolic stress conditions (20 mmol/l glucose, 1.5 mmol/l sodium palmitate, or a combination of 20 mmol/l glucose and 1.5 mmol/l sodium palmitate) compared with the amount of amyloid formed in SC-islets cultured at 5.5 mmol/l glucose (Fig. 2c, d). Moreover, electron microscopy examination of the SC-islets after the metabolic stress culture conditions revealed the presence of amyloid (Fig. 2e). In addition, the dense core insulin granules identified in SC-islets cultured in 5.5 mmol/l glucose were diminished in SC-islets cultured under metabolic stress conditions (Fig. 2e). Thus, we successfully established an in vitro SC-islet model for IAPP amyloid formation that can be manipulated, and this model can be used to study the potential targets that can prevent amyloid formation. As high levels of glucose stimulate amyloid formation in both human islets and SC-islets, only 20 mmol/l glucose was used as the metabolic stress condition in the subsequent experiments.

### Overexpression of Bri2 BRICHOS domain in SC-islets

To find the potential way to inhibit IAPP amyloid formation in SC-islets, Bri2 BRICHOS domain, an endogenous protein peptide with chaperone activity, was studied. Bri2 is proteolytically cleaved during the passage through the secretory pathway. By processing by ADAM metallopeptidase domain 10 (ADAM10), BRICHOS domain is released from Bri2 into the luminal side of the endoplasmic reticulum Golgi site pathway. This enables the BRICHOS domain to be shed into extracellular space [35]. By searching for the online available RNA-seq and genome-wide genotyping data of human islets https://mae.crc.med.lu.se/IsletGeneView/e (accessed 12 January 2023) [32], we found that *ITM2B* was highly expressed in human islets; its expression was positively correlated to *INS* gene expression but negatively correlated to *IAPP* gene expression.

**Fig. 3:**
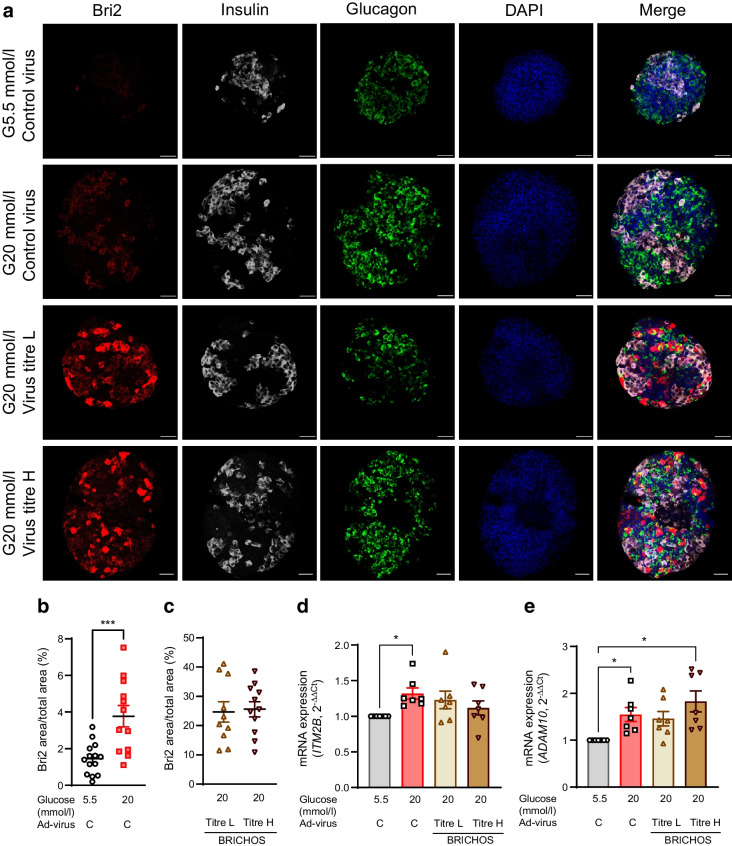
Overexpression of Bri2 BRICHOS domain in SC-islets. SC-islets were transduced with the Ad-control (Ad-virus C) or Ad-BRICHOS virus with low or high titre (Ad-virus BRICHOS titre L and titre H, respectively) for 30 h followed by culturing under normal glucose (G5.5 mmol/l) or high glucose (G20 mmol/l) for 7 days. (**a**–**c**) After culture, the expression levels of Bri2 in SC-islets were confirmed by immunostaining and the representative images are shown (**a**), and the ratio of Bri2 positive area to total SC-islet area in control (**b**) and BRICHOS-overexpressing (**c**) SC-islets was calculated and presented (Bri2, red; insulin, grey; glucagon, green; and DAPI, blue). Scale bar, 50 µm. *n*=total of 12–18 SC-islets from four different batches of differentiations (3–5 SC-islets per differentiation). (**d**, **e**) The gene expression of *ITM2B* (**d**) and *ADAM10* (**e**) was examined by qRT-PCR and data were normalised by using the geometric average C_t_ value of two endogenous controls, *RPS7* and *ACTB*, followed by expression as ${2}^{{-\Delta \Delta \text{C}}_{\text{t}}}$. Results are expressed as mean ± SEM of seven batches of SC-islet differentiations. *p < 0.05, ***p < 0.001, as indicated

Immunolabelling with an anti-Bri2 antibody revealed a low endogenous expression of Bri2 in SC-islets cultured in 5.5 mmol/l glucose and a higher expression in SC-islets cultured in 20 mmol/l glucose for 7 days (Fig. 3a, b). Culture of SC-islets in 20 mmol/l glucose for 7 days increased the mRNA expression level of endogenous *ITM2B* and *ADAM10*, and this increase was not affected by exogenous transduction of the Ad-BRICHOS domain regardless of the virus titre (Fig. 3d, e). At the normal glucose condition (5.5 mmol/l), low and high titres of Ad-BRICHOS did not affect gene expression of *ITM2B* or *ADAM10* (ESM Fig. 1a, b). This observation indicates that under metabolic stress (e.g. high levels of glucose), endogenous Bri2 expression in beta cells was upregulated, and this was paralleled by an increased expression of *ADAM10* that facilitates a proteolytic cleavage and releases the BRICHOS domain. Overexpression of the Bri2 BRICHOS domain in SC-islets was achieved by virus transduction using adenovirus. Virus transduction efficacy ranged between 13% and 69% for the low virus titre and between 31% and 67% for the high virus titre, respectively. As expected, Ad-BRICHOS-transduced SC-islets exhibited a strong signal, confirming the production of exogenous Bri2 BRICHOS domain (Fig. 3a). Immunolabelling for insulin and glucagon was also performed on transduced SC-islets, and the Bri2 BRICHOS domain signal was present in both insulin-positive beta cells and glucagon-positive alpha cells. The virus titre used for Ad-BRICHOS transduction did not affect the number of Bri2-positive cells detected (Fig. 3a, c). Thus, exogenous expression of the Bri2 BRICHOS domain was successfully introduced into the SC-islets.

### Overexpression of Bri2 BRICHOS domain inhibits in vitro IAPP amyloid formation and improves beta cell function in SC-islets

**Fig. 4:**
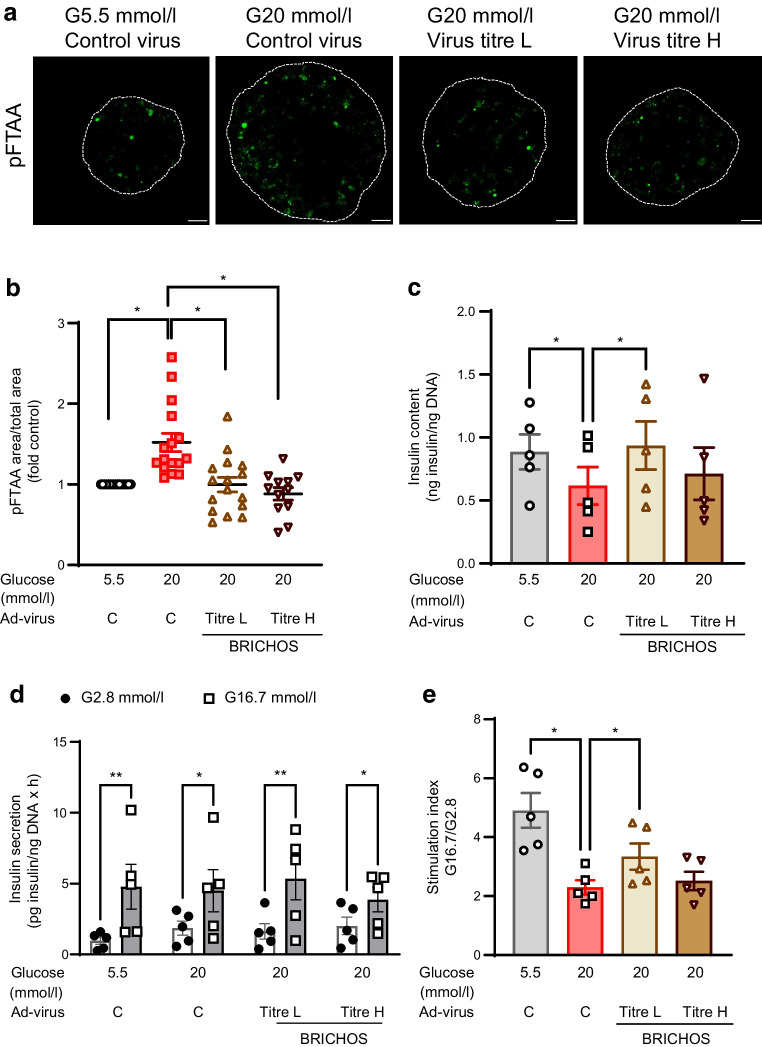
Overexpression of Bri2 BRICHOS domain in SC-islets inhibits in vitro amyloid formation and improves beta cell function under high levels of glucose exposure. SC-islets were transduced with the Ad-control (Ad-virus C) or Ad-BRICHOS virus with low or high titre (Ad-virus BRICHOS titre L and titre H, respectively) for 30 h, followed by culturing under normal glucose (G5.5 mmol/l) or high glucose (G20 mmol/l) for 7 days. (**a**) The amount of amyloid formed after treatment was determined by staining with pFTAA and representative images are shown. Scale bar, 20 µm. (**b**) The ratio of pFTAA-positive area to the total area of SC-islets was calculated from (**a**) and the results were presented as the fold of G5.5 mmol/l group. Results are expressed as mean ± SEM of *n*=total of 12–17 SC-islets from four batches of differentiations (2–6 SC-islets per differentiation). (**c**) Total intracellular insulin content was normalised to total DNA content. (**d**, **e**) Static GSIS was performed in SC-islets cultured with 2.8 mmol/l glucose and 16.7 mmol/l glucose for 60 min. Insulin secreted from static GSIS was normalised to total DNA (**d**) and insulin stimulation index at 7 days of culture was calculated (**e**). Results are expressed as mean ± SEM of five batches of SC-islet differentiations. *p < 0.05, **p<0.01, as indicated

Considering the cytotoxic effects of amyloid formation in human islets [2, 3, 36] and the protective role of the Bri2 BRICHOS domain against amyloid-induced cell death [21], it is attractive to explore whether overexpression of the Bri2 BRICHOS domain in SC-islets can protect beta cell function against glucotoxicity. We found that the amyloid formation induced in SC-islets after 7 days of culture in 20 mmol/l glucose was reduced by overexpression of the Bri2 BRICHOS domain regardless of low or high titres of the Ad-BRICHOS (Fig. 4a, b). However, the decreased intracellular insulin content in SC-islets under long-term high glucose culture could be restored by low but not high titres of Ad-BRICHOS transduction (Fig. 4c). Furthermore, the overexpression of Bri2 BRICHOS domain with a low titre of Ad-BRICHOS virus transduction had a similar protective effect on beta cell function, which was assessed by stimulation index from the static GSIS (Fig. 4d, e).

**Fig. 5:**
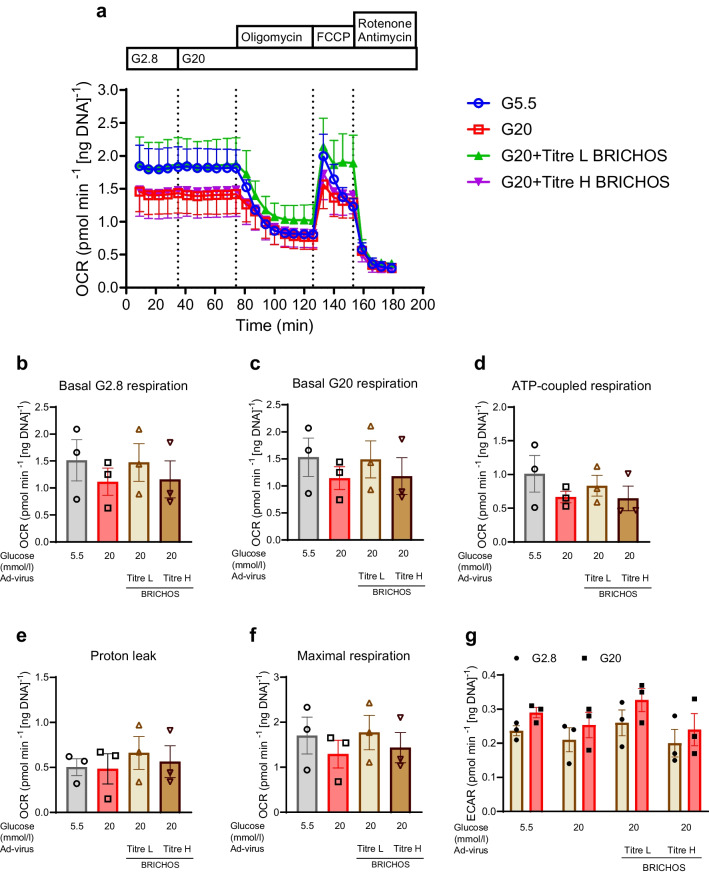
Overexpression of Bri2 BRICHOS domain in SC-islets does not impair mitochondrial respiration. SC-islets were transduced with Ad-BRICHOS virus with low or high titre (Ad-virus BRICHOS titre L and titre H, respectively) for 30 h, followed by culturing under normal glucose (G5.5 mmol/l) or high glucose (G20 mmol/l) for 10 days. After culture, OCR and ECAR were measured. (**a**) The dynamic curve of OCR is shown. The injection of 20 mmol/l glucose, 5 μmol/l oligomycin, 5 μmol/l FCCP, and a combination of 5 μmol/l of rotenone and 5 μmol/l antimycin is shown. (**b**–**g**) Basal mitochondrial OCR at 2.8 mmol/l (**b**) and 20 mmol/l (**c**) was calculated by subtracting non-mitochondrial OCR from the total OCR. The portions of ATP-coupled (**d**) and proton leak OCR (**e**) were calculated based on the oligomycin effect. Maximal OCR was calculated from the FCCP effect (**f**). ECAR measurements were assessed in parallel (**g**). Results are expressed as mean ± SEM of three batches of SC-islet differentiations. Data were analysed for statistical significance using a one-way ANOVA with a Holm–Sidak post hoc test, and no differences were found. FCCP, carbonyl cyanide-*p*-trifluoromethoxyphenylhydrazone

**Fig. 6:**
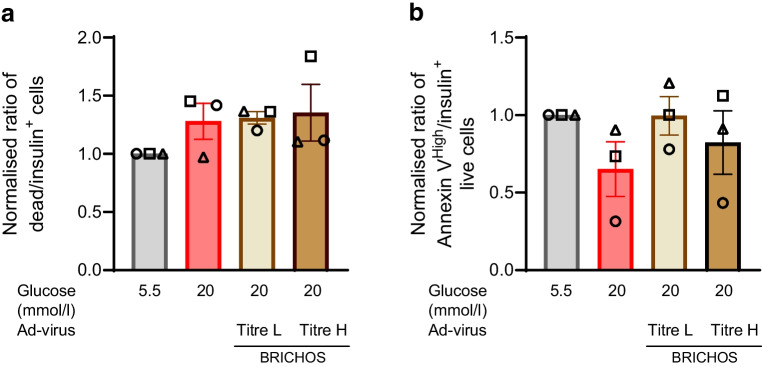
Overexpression of Bri2 BRICHOS domain in SC-islets does not induce beta cell apoptosis. SC-islets were transduced with Ad-BRICHOS virus with low or high titre (Ad-virus BRICHOS titre L and titre H, respectively) for 30 h, followed by culturing under normal glucose (5.5 mmol/l) or high glucose (20 mmol/l) for 10 days. After culture, SC-islet single-cell suspensions were generated, stained and analysed by flow cytometry. Samples were gated for singlets, to exclude debris, and insulin^+^ to identify beta cells. Data were normalised to the control culture conditions for each experiment; circles, squares and triangles each represent a separate experimental replicate. (**a**) Normalised ratio of dead beta cells following culture. (**b**) Normalised ratio of Annexin V^High^ cells among live beta cells. Results are expressed as mean ± SEM of three batches of SC-islet differentiations. Data were analysed for statistical significance by a one-way ANOVA with a Tukey post-test for the ratio of dead beta cells and a Dunn post-test for the ratio of Annexin V^High^ live beta cells, and no differences were found

The overexpression of virus Ad-BRICHOS in SC-islets did not affect the mitochondrial respiration (basal respiration, ATP-coupled respiration and maximal respiration) or glycolysis (basal ECAR) compared with SC-islets treated with 20 mmol/l glucose (Fig. 5a–g). SC-islets transduced with a low titre of Ad-BRICHOS virus showed a similar OCR and ECAR profile as the control SC-islets (Fig. 5a–g). Exposure of the SC-islets to 20 mmol/l glucose revealed a small non-significant (*p*=0.5–0.8) increase in beta cell death but slightly reduced early stage apoptosis marked by Annexin V^High^ staining, but this was not significantly changed with Bri2 BRICHOS overexpression (Fig. 6a, b). This suggests that when treated with 20 mmol/l glucose, beta cells in the SC-islets progress more rapidly from early stage apoptosis to completed apoptosis. Based on these findings, we confirmed that the metabolic stress in vitro in terms of long-term culture in high levels of glucose could trigger amyloid formation in SC-islets and impair beta cell function. Inhibiting amyloid formation in SC-islets via proper overexpression of the Bri2 BRICHOS domain can partially protect beta cell function of SC-islets from metabolic stress induced by long-term exposure to high levels of glucose.

### Overexpression of the Bri2 BRICHOS domain did not affect *IAPP* expression in SC-islets

**Fig. 7:**
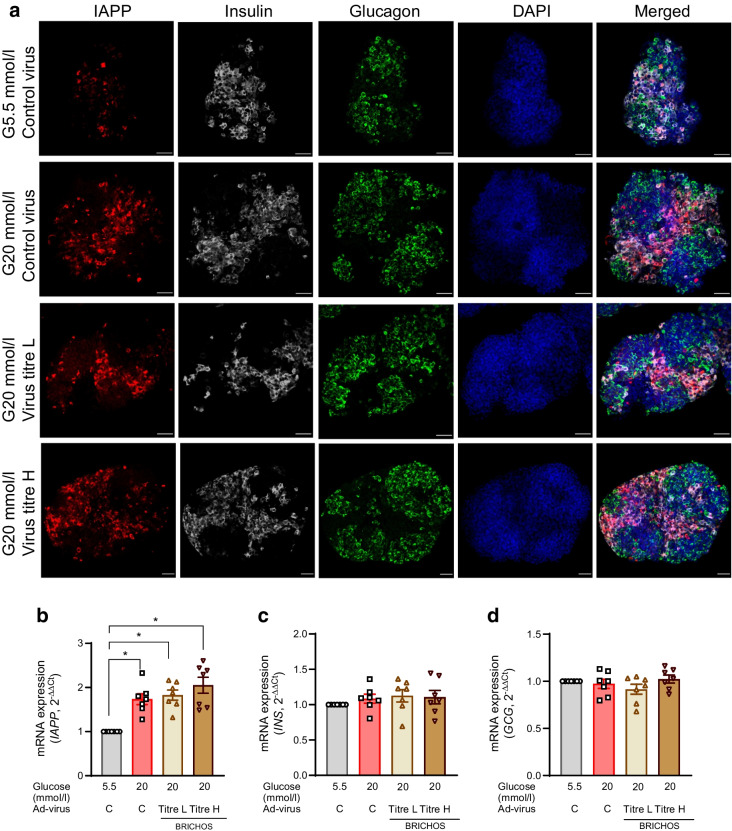
IAPP expression in SC-islets with Bri2 BRICHOS domain overexpression. SC-islets were transduced with the Ad-control (Ad-virus C) or Ad-BRICHOS virus with low or high titre (Ad-virus BRICHOS titre L and titre H, respectively) for 30 h followed by culturing under normal glucose (G5.5 mmol/l) or high glucose (G20 mmol/l) for 7 days. (**a**) After culture, the expression levels of IAPP in SC-islets were evaluated by immunostaining and representative images are shown (IAPP, red; insulin, grey; glucagon, green; and DAPI, blue). Scale bar, 50 µm. *n*=12–15 SC-islets from three different batches of differentiation. (**b**–**d**) The gene expression of *IAPP* (**b**), *INS* (**c**) and *GCG* (**d**) was determined by qRT-PCR. Data were normalised by using the geometric average C_t_ value of two endogenous controls (*RPS7* and *ACTB*), and expressed as ${2}^{{-\Delta \Delta \text{C}}_{\text{t}}}$. Results are expressed as mean ± SEM of seven batches of SC-islet differentiations. *p < 0.05 as indicated

Amyloid forms rapidly in cultured human islets [37] and the formation of amyloid in vitro is glucose- and time-dependent [34]. In line with this observation, the expression of IAPP in SC-islets was triggered by a 7 day exposure to 20 mmol/l glucose (Fig. 7a, b). The overexpression of the Bri2 BRICHOS domain in SC-islets did not affect the induced *IAPP* mRNA expression in response to high glucose culture regardless of the virus or virus titre (Fig. 7b). Thus, the reduction in amyloid formation in high glucose-cultured SC-islets by Bri2 BRICHOS domain overexpression (Fig. 2a, b) was not due to a decreased expression of *IAPP*. Despite the restored insulin content in 20 mmol/l glucose-cultured SC-islets by low titre Ad-BRICHOS transduction, the gene expression of *INS* and *GCG* was unaffected by culture in 20 mmol/l glucose for 7 days regardless of Ad-BRICHOS transduction (Fig. 7c, d). Additionally, after culture in 5.5 mmol/l glucose, low and high titres of Ad-BRICHOS did not affect gene expression of *IAPP*, *INS* or *GCG* (ESM Fig. 1c–e).

## Discussion

In this study, we present our finding that culture under long-term metabolic stress conditions in vitro triggers cytotoxic IAPP amyloid formation in SC-islets. The induced amyloid formation in SC-islets under long-term exposure to high levels of glucose could be ameliorated by the proper overexpression of the Bri2 BRICHOS domain via adenovirus transduction, which could partially contribute to the improved beta cell function in SC-islets under metabolic stress. Moreover, the induced endogenous gene expression of *ITM2B*, *ADAM10* and *IAPP* in SC-islets by long-term culture at a high glucose concentration was unaffected by the overexpression of the Bri2 BRICHOS domain regardless of the adenovirus titre.

Our previous findings confirm IAPP amyloid formation in SC-islets transplanted beneath the kidney capsule 3 months post transplantation in immunodeficient mice [15]. Based on this observation, the present study was performed to investigate under which in vitro circumstances IAPP amyloid forms in SC-islets and also to explore whether the inhibition of amyloid formation in SC-islets can improve beta cell function. As a first step, we aimed to characterise the similarities between SC-islets and human islets regarding the *IAPP* expression and distribution. Despite the slightly different proportions of insulin-secreting beta cells and glucagon-secreting alpha cells, the expression of *IAPP* was similar between human islets and SC-islets. Moreover, IAPP was shown to be colocalised with insulin-positive beta cells but not glucagon-positive alpha cells by immunostaining, suggesting that IAPP is located in SC-islet beta cells, as in human islet beta cells where both IAPP and insulin were packaged together in the insulin secretory granules [3, 38]. It has been reported that the SC-islets differentiated from the hPSCs in vitro could not completely recapitulate the in vivo maturation of adult human islets [14, 15]. However, IAPP was recently identified as a potential marker for a more mature subpopulation of SC-beta cells [39], suggesting that the SC-islets generated in this study contained mature beta cells and can be used to test the development of amyloid formation in in vitro experiments.

Insulin resistance and hyperglycaemia may contribute to the formation of amyloid in human islets in vivo by the increased production of IAPP from the beta cells [40, 41]. There is a strong association between the degree of amyloid deposition in individuals with type 2 diabetes and the presence of beta cell apoptosis and reduced beta cell mass [2]. In human islets, amyloid is found both intra- and extracellularly, caused by two pathways that occur side by side but that are dependent on each other [36]. Extensive intracellular amyloid results in cell death [2, 42], and amyloid escaping degradation by macrophages [43] can seed amyloid growth of IAPP secreted from the surrounding beta cells. In amyloid toxicity, oligomers are described as cytotoxic species [44], while mature fibrils will affect the architecture of the islet and interfere with hormone secretion [3]. In this study, we confirmed that in vitro cultured SC-islets could develop IAPP amyloid formation under long-term exposure to different metabolic stress circumstances (e.g. high levels of glucose, sodium palmitate and glucose plus sodium palmitate), as assessed by staining with the amyloid-specific ligand pFTAA. This was consistent with the findings in human islets in in vitro culture where IAPP amyloid was formed under metabolic stress and played an important role in beta cell death [2, 42, 45]. The induced IAPP amyloid formation also contributed to the impaired beta cell function in SC-islets in terms of GSIS and intracellular insulin content, although with no change in the mRNA expression level of the *INS* gene.

Human IAPP is unusually amyloidogenic, yet IAPP remains as non-aggregated in most healthy individuals, suggesting the presence of an endogenous inhibitor of IAPP aggregation in the secretory granules of beta cells where high concentrations of IAPP and insulin are stored [3]. Even though insulin was shown to prevent IAPP aggregation in a concentration-dependent manner in vitro [46], the mature peptides of IAPP and insulin do not interact with each other due to the differences in their intragranular localisation (i.e. IAPP is in the halo region of the granule and insulin together with Zn^2+^ makes up the dense core region) [38]. This observation led to the search for other endogenous molecules with inhibitory effects on IAPP fibrillation*.* In this study, the role of the Bri2 BRICHOS domain, an endogenous protein with chaperone activity, was studied for the first time in relation to beta cell function in SC-islets. *ITM2B* was shown to be positively correlated to *INS* while negatively correlated to *IAPP* gene expression from an online database [32], indicating that Bri2 probably plays a protective role in human islet beta cells. In SC-islets, exposure to metabolic stress increases the expression of endogenous Bri2 at protein and mRNA levels and *ADAM10* gene expression, which is expected to increase the release of the BRICHOS domain from Bri2. It is possible that the increased expression of Bri2 was a compensatory mechanism but it remains insufficient to prevent the aggregation of IAPP, which also increased in response to metabolic stress. On the other hand, when the Bri2 BRICHOS domain was overexpressed in SC-islets through adenovirus transduction and confirmed with immunostaining with a Bri2 antibody, we observed that the low virus titre was sufficient for almost complete prevention of amyloid in SC-islets, without causing any negative effect on IAPP expression. In vitro, 5 μmol/l Bri2 BRICHOS prevents aggregation of 10 μmol/l IAPP, an inhibition lost at higher molar ratios [21]. This suggests that an adequate level of Bri2 BRICHOS is crucial for inhibiting amyloid formation. Chaperones are often general, working on a broad range of protein targets. Bri2/BRICHOs prevents amyloid formation from both IAPP (in islets) and Aβ (amyloid protein that deposits in patients with Alzheimer’s disease). Therefore, a higher virus titre does not necessarily mean more protection; instead, the optimal effect is obtained when the necessary molar ratio of IAPP and BRICHOS is reached. The absence of further improvement after high virus titre transduction indicates that low virus titre transduction was sufficient to reach an adequate concentration. Furthermore, it may contribute to the restoration of insulin content and improved GSIS in SC-islets under metabolic stress.

Early nomenclature describes amyloid as extracellular deposits, and new therapies include antibodies against amyloid oligomers or mature fibrils. Lecanemab, a monoclonal antibody that binds with high affinity to soluble amyloid beta (Aβ) protofibrils, was recently approved by the US Food and Drug Administration (FDA) [47]. Administration of lecanemab reduces the extracellular amyloid load and slows the cognitive decline in individuals with early Alzheimer’s disease. In an early study on islet amyloid prevention performed on islets isolated from human IAPP-expressing mice, toxic oligomers were present in beta cells, and administration of oligomer-specific antibodies did not affect beta cell apoptosis [42]. Instead, the treatment strategy should prevent the formation of intracellular aggregates and thereby avert cytotoxicity. Bri2 BRICHOS has been shown to prevent amyloid fibril formation from IAPP and Aβ in vitro [48, 49] and i.v. injections of BRICHOS reduced the amyloid load and gliosis in an mouse model of Alzheimer’s disease [50]. The results confirm the effect of BRICHOS on protein aggregation and also on the clearance of formed amyloid. Bri2/BRICHOS is of particular interest as beta cells already express this protein. Therefore, one should focus on regulating the expression of the endogenous *ITM2B* gene or increasing the processing of Bri2 to increase the production of BRICHOS. In islet transplantation, overexpression of BRICHOS could be obtained with viral transduction.

Certain limitations of this study should be acknowledged. First, there are some experiments wherein the number of observations was relatively low. However, the well-controlled differentiation protocol and the quality control that SC-islets underwent before inclusion in a study ensured low batch variation. In addition, the impact of Bri2 BRICHOS on preventing cytotoxic IAPP amyloid is clearly visible. Second, the transduction efficiency between batches of SC-islets varies and is challenging to quantify with high precision. One explanation could be that not all transduced cells may express Bri2 at the same level, leading to a variability in the staining intensity. Moreover, our SC-islets also varied in size and density, which could further impact the transduction efficiency. This could impose an additional translational challenge. Verification of our findings in an in vivo model is required.

In conclusion, we have demonstrated that Bri2 colocalises with insulin in SC-islet beta cells. Furthermore, overexpression of the Bri2 BRICHOS domain can prevent amyloid formation and improve beta cell function in SC-islets exposed to metabolic stress. This work emphasises the importance of regulating IAPP in the prevention of amyloid and opens up the potential for the development of Bri2 BRICHOS domain-directed targets in SC-islets to improve the function of beta cells.

## Supplementary Information

Below is the link to the electronic supplementary material.

ESM (PDF 532 KB)

## Data Availability

All data supporting the findings of this study are available within the paper and its ESM. Raw data are available on request from the authors.

## Abbreviations

Aβ: Amyloid β
ADAM10: ADAM metallopeptidase domain 10
ECAR: Extracellular acidification rate
GSIS: Glucose-stimulated insulin secretion
hPSC: Human pluripotent stem cell
IAPP: Islet amyloid polypeptide
IGW: Islet Gene View
ITM2B: Integral membrane protein 2B
OCR: Oxygen consumption rate
pFTAA: Pentameric formyl thiophene acetic acid
qPCR: Quantitative real-time PCR
qRT-PCR: Quantitative reverse transcription PCR
SC-islet: Stem cell-derived islet
