# Supplementary material for: Bri2 BRICHOS domain inhibits IAPP amyloid formation and improves beta cell function in stem cell-derived islets under metabolic stress
Source: Diabetologia. 2025 Nov 10;69(2):436–50. doi: 10.1007/s00125-025-06582-5 (PMC12779682; doi:10.1007/s00125-025-06582-5)
Supplement: Supplementary file 1 — ESM (PDF 532 KB) [file 125_2025_6582_MOESM1_ESM.pdf]

Electronic Supplementary Material (ESM)

ESM Table 1. Donor information for human islets used in this study.

Donor number	Age (year)	Gender	BMI (kg/m^2)	HbA1c (mmol/mol)
1	72	Male	24.5	39.0 (5.7 %)
2	58	Male	21.8	35.0 (5.4 %)
3	67	Female	25.8	35.0 (5.4 %)
4	65	Female	23.8	47.0 (6.5 %)
5	59	Female	35.2	37.0 (5.5 %)
6	62	Female	25.5	42.0 (6.0 %)
7	42	Male	30.0	39.0 (5.7 %)
8	69	Male	27.1	N/A
9	69	Male	31.1	40.0 (5.8 %)
10	64	Female	24.6	45.0 (6.3 %)

N/A: Not available

ESM Table 2. Primers for qRT-PCR experiments.

Gene name	MIM	Forward primer (5’-3’)	Reverse primer (5’-3’)
ADAM10	602192	GAGGAGTGTACGTGTGCCAG	GTTCGACCACTGAAGTGCCT
IAPP	147940	AGCGGAAATGCAACACTGCCAC	CGTTGGTAGATGAGAGAATGGCA
INS	176730	ACGAGGCTTCTTCTACACACCC	TCCACAATGCCACGCTTCTGCA
ITM2B	603904	AGAAGAGCCTGGTGTTGGTGCA	CCACAGTAGTACACGTCATCTGG
GCG	138030	CGTTCCCTTCAAGACACAGAGG	ACGCCTGGAGTCCAGATACTTG
ACTB	102630	CACCATTGGCAATGAGCGGTTC	AGGTCTTTGCGGATGTCCACGT
GUSB	611499	ATGCCATCGTGTGGGTGAAT	TGGCGATAGTGATTCGGAGC
RPS7	603658	GAAGTTGGTGGTGGTCGGAA	TGCACAGCTGTCAGAGTACG

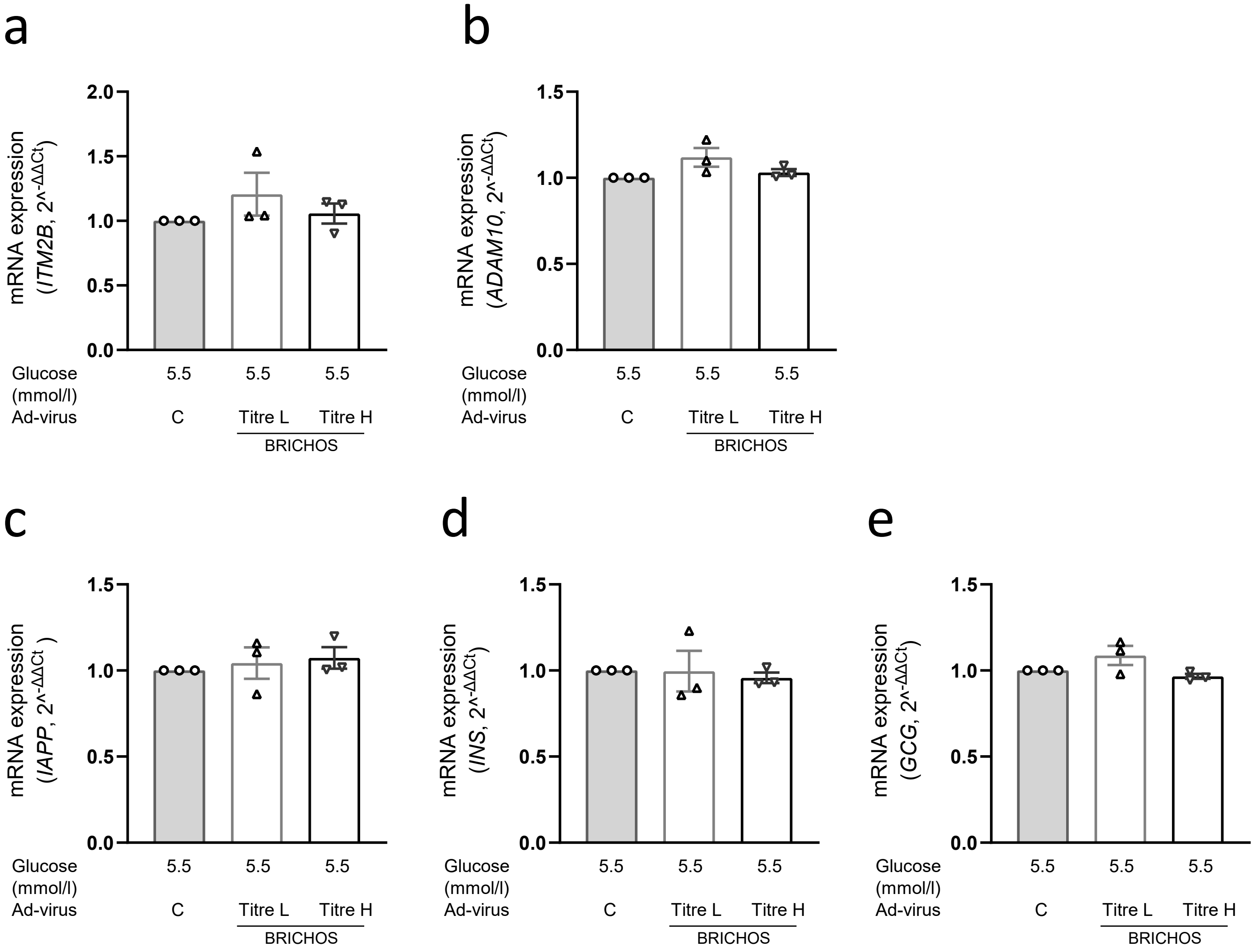

ESM Figure 1. **Gene expression in stem cell-derived islets (SC-islets) with Bri2 BRICHOS domain overexpression at 5.5 mmol/l glucose.**

SC-islets were transduced with the control virus (Ad-virus C) or Ad-BRICHOS virus with low or high titre (Ad-virus BRICHOS titre L and titre H, respectively) for 30 hours followed by culturing under normal glucose (G5.5 mmol/l) for 7 days. After treatment, the gene expression of *ITM2B* (**a**), *ADAM10* (**b**), *IAPP* (**c**), *INS* (**d**) and *GCG* (**e**) was performed by qRT-PCR. Data were normalised by using the geometric average Ct value of two endogenous controls *RPS7* and *ACTB*, respectively, and expressed as $2^{-\triangle\triangle Ct}$. Results are expressed as mean ± SEM of 3 batches of SC-islet differentiations.

Checklist for reporting human islet preparations used in research

Adapted from Hart NJ, Powers AC (2018) Progress, challenges, and suggestions for using human islets to understand islet biology and human diabetes. Diabetologia https://doi.org/10.1007/s00125-018-4772-2

Islet preparation	1	2	3	4	5	6	7	8[a]
**MANDATORY INFORMATION**								
Unique identifier	H2550	H2555	H2565	H2691	H2681	H2692	Isolate date 202302	H2614
Donor age (years)	72	58	67	65	59	62	42	69
Donor sex (M/F)	M	M	F	F	F	F	M	M
Donor BMI (kg/m^2)	24.5	21.8	25.8	23.8	35.2	25.5	30.0	27.1
Donor HbA_{1c} or other measure of blood glucose control	39	35	35	47	37	42	39	N/A
Origin/source of islets[b]	NNCIT	NNCIT	NNCIT	NNCIT	NNCIT	NNCIT	Prodo	NNCIT
Islet isolation centre	Uppsala	Uppsala	Uppsala	Uppsala	Uppsala	Uppsala	Prodo	Uppsala
Donor history of diabetes? Please select yes/no from drop down list	No	No	No	No	No	No	No	No
**If Yes, complete the next two lines if this information is available**								
Diabetes duration (years)								
Glucose-lowering therapy at time of death[c]								
**RECOMMENDED INFORMATION**								
Donor cause of death								
Warm ischaemia time (h)								
Cold ischaemia time (h)								

Estimated purity (%)								
Estimated viability (%)								
Total culture time (h)[d]								
Glucose-stimulated insulin secretion or other functional measurement[e]								
Handpicked to purity? Please select yes/no from drop down list								
Additional notes								

[a]If you have used more than eight islet preparations, please complete additional forms as necessary
[b]For example, IIDP, ECIT, Alberta IsletCore
[c]Please specify the therapy/therapies
[d]Time of islet culture at the isolation centre, during shipment and at the receiving laboratory
[e]Please specify the test and the results

Diabetologia

Checklist for reporting human islet preparations used in research

Adapted from Hart NJ, Powers AC (2018) Progress, challenges, and suggestions for using human islets to understand islet biology and human diabetes. Diabetologia https://doi.org/10.1007/s00125-018-4772-2

**Islet preparation**	**9**	**10**						
**MANDATORY INFORMATION**								
Unique identifier	H2603	H2545						
Donor age (years)	69	64						
Donor sex (M/F)	M	F						
Donor BMI (kg/m^2)	31.1	24.6						
Donor HbA_{1c} or other measure of blood glucose control	40	45						
Origin/source of islets[b]	NNCIT	NNCIT						
Islet isolation centre	Uppsala	Uppsala						
Donor history of diabetes? Please select yes/no from drop down list	No	No						
**If Yes, complete the next two lines if this information is available**								
Diabetes duration (years)								
Glucose-lowering therapy at time of death[c]								
**RECOMMENDED INFORMATION**								
Donor cause of death								
Warm ischaemia time (h)								
Cold ischaemia time (h)								

Estimated purity (%)								
Estimated viability (%)								
Total culture time (h)[d]								
Glucose-stimulated insulin secretion or other functional measurement[e]								
Handpicked to purity? Please select yes/no from drop down list								
Additional notes								

[a]If you have used more than eight islet preparations, please complete additional forms as necessary
[b]For example, IIDP, ECIT, Alberta IsletCore
[c]Please specify the therapy/therapies
[d]Time of islet culture at the isolation centre, during shipment and at the receiving laboratory
[e]Please specify the test and the results
